# Enhancing innate antiviral immune responses in rainbow trout by double stranded RNA delivered with cationic phytoglycogen nanoparticles

**DOI:** 10.1038/s41598-019-49931-2

**Published:** 2019-09-20

**Authors:** Tamiru N. Alkie, Jondavid de Jong, Kristof Jenik, Karl M. Klinger, Stephanie J. DeWitte-Orr

**Affiliations:** 1 Department of Health Sciences, Wilfrid Laurier University, Waterloo, ON Canada; 2 Glysantis Inc., Guelph, ON Canada

**Keywords:** Cell signalling, Pattern recognition receptors, Virus-host interactions

## Abstract

Innate immunity is induced when pathogen-associated molecular patterns (PAMPs) bind host pattern recognition receptors (PRRs). Polyinosinic:polycytidylic acid [poly(I:C)] is a synthetic analogue of viral dsRNA that acts as a PAMP, inducing type I interferons (IFNs) in vertebrates. In the present study, the immunostimulatory effects of high molecular weight (HMW) poly(I:C) in rainbow trout cells were measured when bound to a cationic phytoglycogen nanoparticle (Nano-HMW). The physical characteristics of the nanoparticle itself, when bound to different lengths of dsRNA and when cell associated was evaluated. Optimal concentration and timing for innate immune stimulation was measured using the RTG-P1 reporter cell line. The immunostimulatory effects of HMW poly (I:C) was compared to Nano-HMW *in vitro* using the RTgutGC cell line cultured in a conventional monolayer or a transwell culture system. The ability of an activated intestinal epithelium to transmit an antiviral signal to macrophages was evaluated using a co-culture of RTgutGC cells and RTSll (a monocyte/macrophage cell). In all culture conditions, Nano-HMW was a more effective inducer of IFN-related antiviral immune responses compared to HMW poly (I:C) alone. This study introduces the use of cationic phytoglycogen nanoparticles as a novel delivery system for immunomodulatory molecules to enhance immune responses in aquatic vertebrates.

## Introduction

The innate immune system of vertebrates consists of cells armed with pattern recognition receptors (PRRs) that sense pathogen-associated molecular patterns (PAMPs). Double-stranded (ds)RNA is a PAMP produced by almost all viruses during their replication cycle^1^. After recognition of dsRNA by PRRs, the triggered intracellular signaling pathways result in the induction of type I interferons (IFNs) and pro-inflammatory cytokines^2^. While the number and type of IFNs varies between teleosts and mammals^3,4^, the local and systemic antiviral effects of IFNs are similar in both species^5^. The IFN signaling system in fish induces interferon stimulated genes (ISGs), a group of proteins that work together to limit virus replication^6–9^. Two of these ISGs in fish are: myxovirus resistance (Mx) and viral hemorrhagic septicemia virus (VHSV)-induced gene (Vig)-3^10–14^.

The fish intestine, gills and fin bases are the prime route of pathogen entry and represent the first barrier between the fish and its environment. The constituent cells in these tissues have the capability to respond to PAMPs via their PRRs^15–17^, thus the induction of broad-spectrum antiviral responses on mucosal surfaces is an attractive prophylactic strategy for antiviral therapeutics^4^. PAMPs, including the toll-like receptor (TLR)3 ligand polyinosnic:polycytidylic acid [poly(I:C)], have been shown to effectively limit virus replication^12^ and act as adjuvants for prophylactic vaccines^18^ in aquaculture applications. However, one practical limitation of using TLR ligands to control viral infections in fish is the duration and quality of innate immune responses being induced^19^. In recent studies nanoparticle delivery platforms in combination with PAMPs, or their synthetic mimics, have shown promise in inducing efficacious innate immune responses^20^. Strategies to date for application in vertebrate species have focused on using synthetic or natural polymers, lipids, lipid-polymer hybrids and self-assembling compounds to produce nano- or micro-sized delivery systems, whereby TLR ligands are entrapped or adsorbed^21–23^. The current study uses a cationic phytoglycogen nanoparticle (NP) derived from non-GMO sweet corn to deliver high molecular weight (HMW) poly(I:C). This plant-derived phytoglycogen NP is non-toxic and monodispersed product with a dendrimeric structure^24^. It is amenable to chemical modifications, which allow the addition of different structural and functional groups on its surface so that significant amounts of active compounds can be bound to its surfaces. The cationic version of this nanoparticle, similar to cationic polyethylenimine, likely condenses nucleic acid structures on its surface, protecting RNA from degradation and facilitating RNA entry into target cells^25^.

In the current study, we hypothesized that HMW poly(I:C) delivered by cationic phytoglycogen NPs (Nano-HMW) would be a more effective antiviral inducer in rainbow trout cells compared to the free HMW poly(I:C). These formulations were tested using an intestinal epithelial cell line (RTgutGC) cultured in a conventional monolayer and in a transwell culture system that better mimics an intestinal epithelial layer^26,27^. The RTG-P1 reporter cell line, which has an inducible luciferase gene under the control of rainbow trout Mx1 promoter, was used as a rapid means of determining the duration of the induced innate response and for determining optimal doses for subsequent experiments. In order to assess the interactions of intestinal epithelial cells with the underlying antigen presenting cells, RTS11 (a monocyte/macrophage cell line) in combination with RTgutGC cells within the transwell culture system was used. The antiviral responses stimulated by Nano-HMW and HMW poly(I:C) alone were measured by the level of expression of IFN and ISGs transcripts, Mx1 promoter activity, and antiviral protection assays. This is the first study to evaluate innate antiviral responses induced by dsRNA in conjunction with cationic phytoglycogen NPs in fish. Thus, our study presents a mechanism of PAMP delivery to fish that increases immune stimulation and hence represents an avenue for future development of antiviral therapies in an economically relevant fish species.

## Results

### Characterizing the cationic phytoglycogen NP’s physical properties, binding capabilities and cellular association patterns

The determination of the size and surface charge of cationic phytoglycogen NPs are critical quality control parameters for properly characterizing a NP’s formulation. These properties determine the ability for uptake by cells, cellular trafficking and dictate the nature of immunological responses^28–30^ Smaller NPs, in the size ranges of most viruses, are preferentially taken up via clathrin or caveolae-mediated endocytosis and can effectively deliver drugs across biological barriers^29^. Dynamic light scattering analysis indicated that these NPs had an average size of 70 nm in diameter with a net positive surface charge of +25 mV in a liquid non-buffered media when measured at 25 °C. Moreover, the polydispersity index (PDI), which measures the width of size distributions of the particles, indicated a PDI of about 0.1. This suggests that the NPs have a uniform size and do not aggregate.

**Figure 1:**
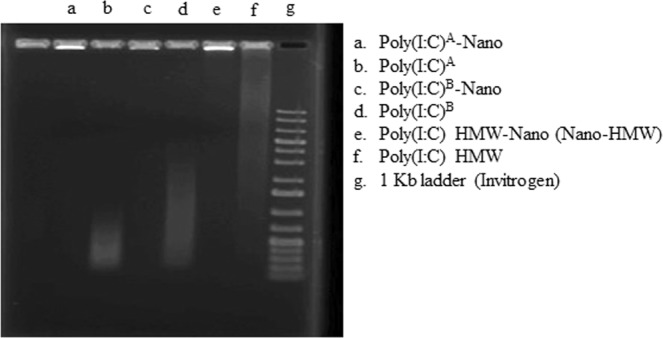
Deducing the binding efficacy of dsRNAs on cationic phytoglycogen NPs using a gel shift assay. Three different dsRNA preparations (poly(I:C)^A^, poly(I:C)^B^ and HMW poly(I:C)) representing different lengths of dsRNA were combined with cationic phytoglycogen NPs for 20 min to form complexes. These complexes were subjected to electrophoresis through a 1% agarose gel. The gel was stained with ethidium bromide and RNA bands were visualized under UV-light. Free dsRNA can be observed running through the gel as a smear, while dsRNA-NP complexes are a bright band stuck within the well. Poly(I:C)^A^ is from Sigma-Aldrich and poly(I:C)^B^ is from (InvivoGen). The superscripts denote the sources of poly(I:C).

Next, it was important to determine the extent by which poly (I:C) was capable of adsorbing onto the cationic phytoglycogen NPs and whether dsRNA length affected binding. This was determined by gel shift assay using three different dsRNA preparations, representing three different average lengths. Specifically, poly(I:C)^A^ had an average length of ~400 bp, poly(I:C)^B^ had an average length of ~750 bp, and HMW poly(I:C) had an average length of ~3000 bp. As indicated in Fig. 1, the dsRNA-NP complexes migrated more slowly than the corresponding free dsRNA. At a weight ratio of 1:1, no free dsRNA could be observed in the wells containing dsRNA and cationic phytoglycogen NPs (Nano-HMW), suggesting ~100% of the dsRNA is bound to the NPs (Fig. 1).

**Figure 2:**
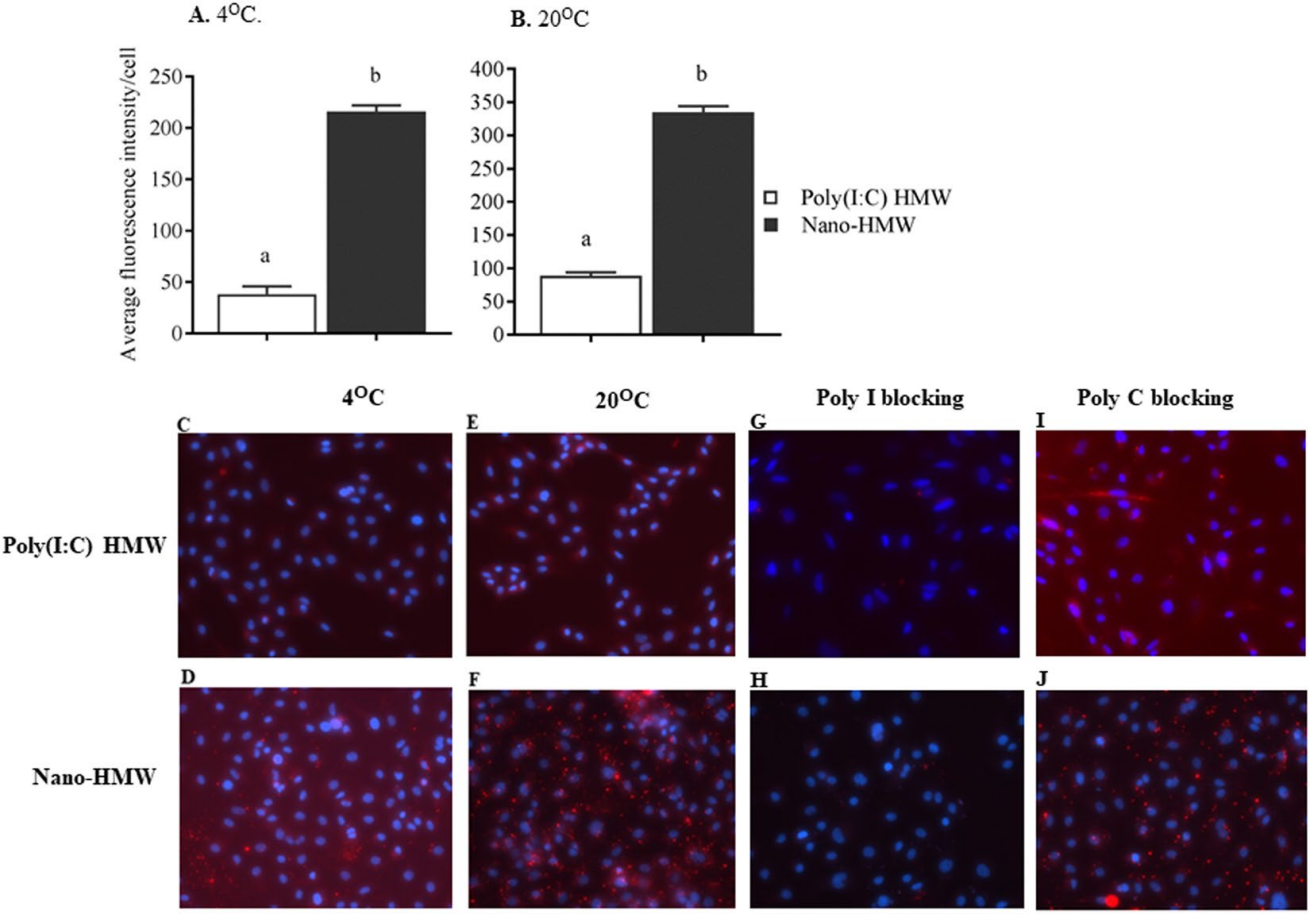
Determining the uptake of HMW poly(I:C) or Nano-HMW by RTgutGC cells incubated at different temperature conditions and in the presence of SR-A competitive and non-competitive ligands. RTgutGC cells were treated with Alexafluor 488 labelled free HMW poly(I:C) or Nano-HMW (500 ng/mL) and incubated at 4 °C and 20 °C for 3 hr. Fluorescence intensity and distribution per cell indicated bound or internalized dsRNA. Fluorescence intensity per cell was quantified at 4 °C (**A**) and 20 °C (**B**). Distribution patterns of fluorescent labeled free HMW poly(I:C) (**C**) and Nano-HMW (**D**) at 4 °C, and free HMW poly(I:C) (**E**) and Nano-HMW (**F**) at 20 °C are depicted. For the blocking assays, RTgutGC cells were pretreated at concentration 200 µg/mL with the SR-A competitive ligand, poly I (**G**,**H**) and its non-competitive counterpart, poly C (**I**,**J**) for 3 hr, which was followed by treatment with HMW poly(I:C) or Nano-HMW (500 ng/mL) in the presence of the ligands for another 3 hr. In all experiments, the extent of uptake was determined by measuring fluorescent intensity per cell using Nikon-NIS-elements software. Data are expressed as mean ± SEM. *P* < 0.05 was considered significant. Different letters within a defined single time point show significant differences between groups. In the fluorescence images, HMW poly(I:C) appears red and cell nuclei appear blue. Magnification 200X.

In order to assess the cell association of poly(I:C) by RTgutGC cells, HMW poly(I:C) was fluorescently labeled. RTgutGC cells were treated with labeled HMW poly(I:C) or labeled HMW poly(I:C) complexed with cationic phytoglycogen NPs (Nano-HMW). In all cases, RTgutGC cells were able to associate with both HMW poly (I:C) and Nano-HMW. More dsRNA was cell associated at 20 °C compared to 4 °C, suggesting an active cellular process of uptake, and there was substantially more dsRNA associated with the cell when the dsRNA was bound to the NP at both 4 °C and 20 °C (Fig. 2A–F). The association with dsRNA was homogenous among the cell population and bound in a punctate fashion as previously shown in murine embryonic fibroblasts and rainbow trout cells^31,32^. Fluorescence imaging suggests that the particles are either cell surface associated or internalized, or a combination of the two. To determine the mechanism of entry, RTgutGC cells were first treated with Poly I, a competitive SR-As ligand, or with Poly C, a non-competitive counterpart. While, Poly I blocked the entry of Nano-HMW (Fig. 2H), Poly C did not (Figure 2J); indicating this NP formulation utilizes SR-As as one of the major cell surface receptors to mediate dsRNA uptake. This trend was also observed with HMW poly (I:C) alone (Fig. 2G,I).

### Determining the optimal treatment parameters for stimulating an antiviral response

**Figure 3:**
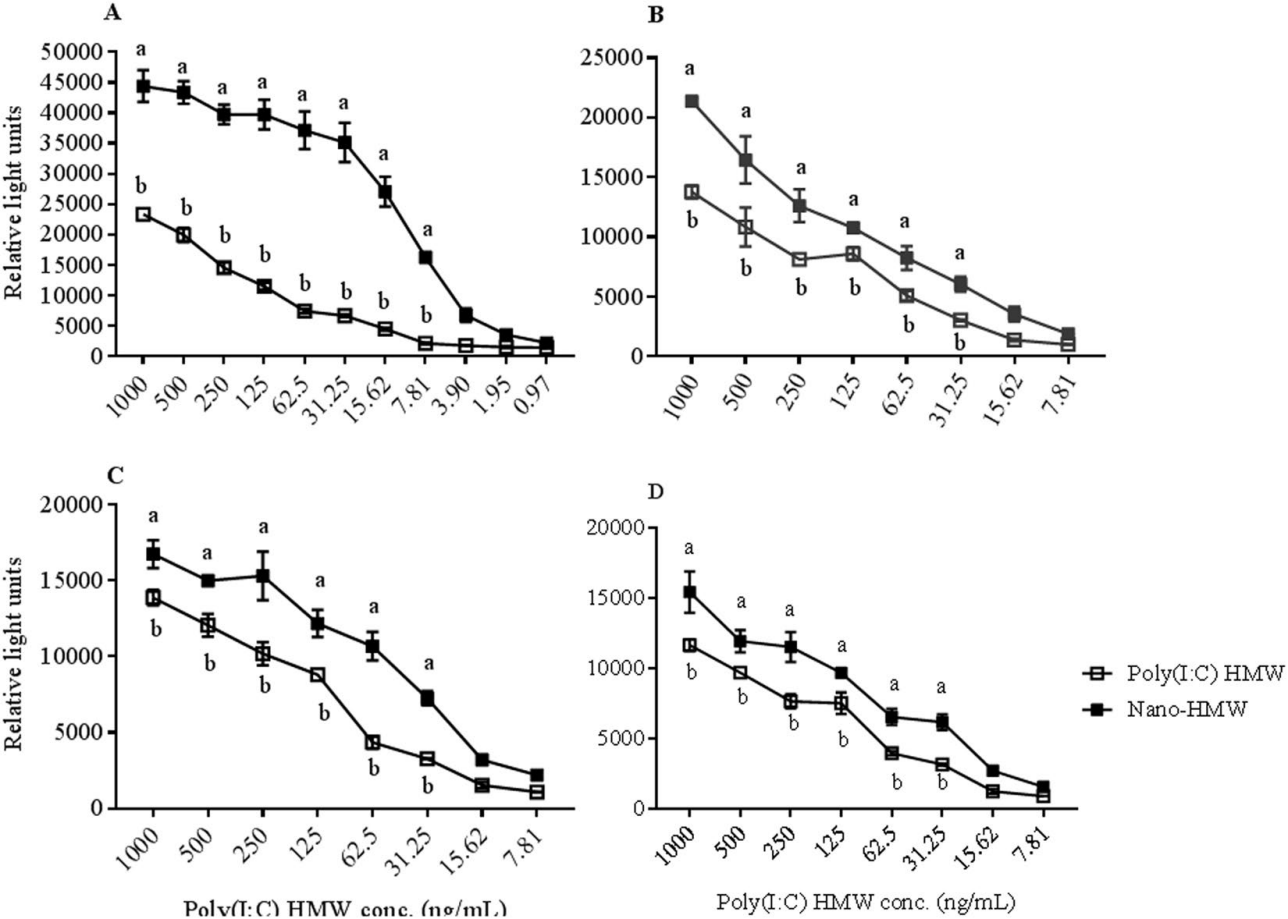
Dose response study of Mx1 promoter activity induced by HMW poly(I:C) and Nano-HMW. RTG-P1, cells expressing firefly luciferase under the control of the rainbow trout Mx1 promoter, were treated with either HMW poly(I:C) or Nano-HMW for 24 hr (**A**), 48 hr (**B**), 72 hr (**C**) and 96 hr (**D**) after which luciferase activity was measured. The data represent the average ± SEM of 3 independent trials, carried out in 96-well black plates. A paired Student’s T-tests were used to analyze data at each concentration; *P* < 0.05 was considered significant. Different letters within a defined single concentration show significant differences between groups at that concentration.

RTG-P1 cells, an Mx1-promoter reporter system, were used to determine the optimal dose and time whereby HMW poly(I:C) induces IFN-related pathways in rainbow trout cells. Nano-HMW treatment consistently induced significantly higher Mx1 promoter activity compared to the corresponding free HMW poly(I:C) 24 hr post treatment at concentrations ranging from 7.8–1000 ng/mL (Fig. 3A), and at 48 hr (Fig. 3B), 72 hr (Fig. 3C) and 96 hr (Fig. 3D) post treatment at concentrations ranging from 31.25–1000 ng/mL. Notably, the medium and Nano-mock alone controls did not enhance luciferase production from that of background. As the induced responses were highest at 24 hr (Fig. 3A) posttreatment, this time point was determined to be the optimal time for induction of the IFN-related response. Based on the 24 hr data, the optimal dosage chosen for subsequent experiments was 62.5 ng/mL as a low dsRNA dose and 1000 ng/mL as a high dsRNA dose. In addition to the 24 h time point, 3 hr and 6 hr post treatment were also chosen for subsequent assays using RT-qPCR, as this method of quantification is more sensitive to changes at earlier time points.

### Cationic phytoglycogen NPs enhances the immuno-stimulatory effects of HMW poly(I:C)

**Figure 4:**
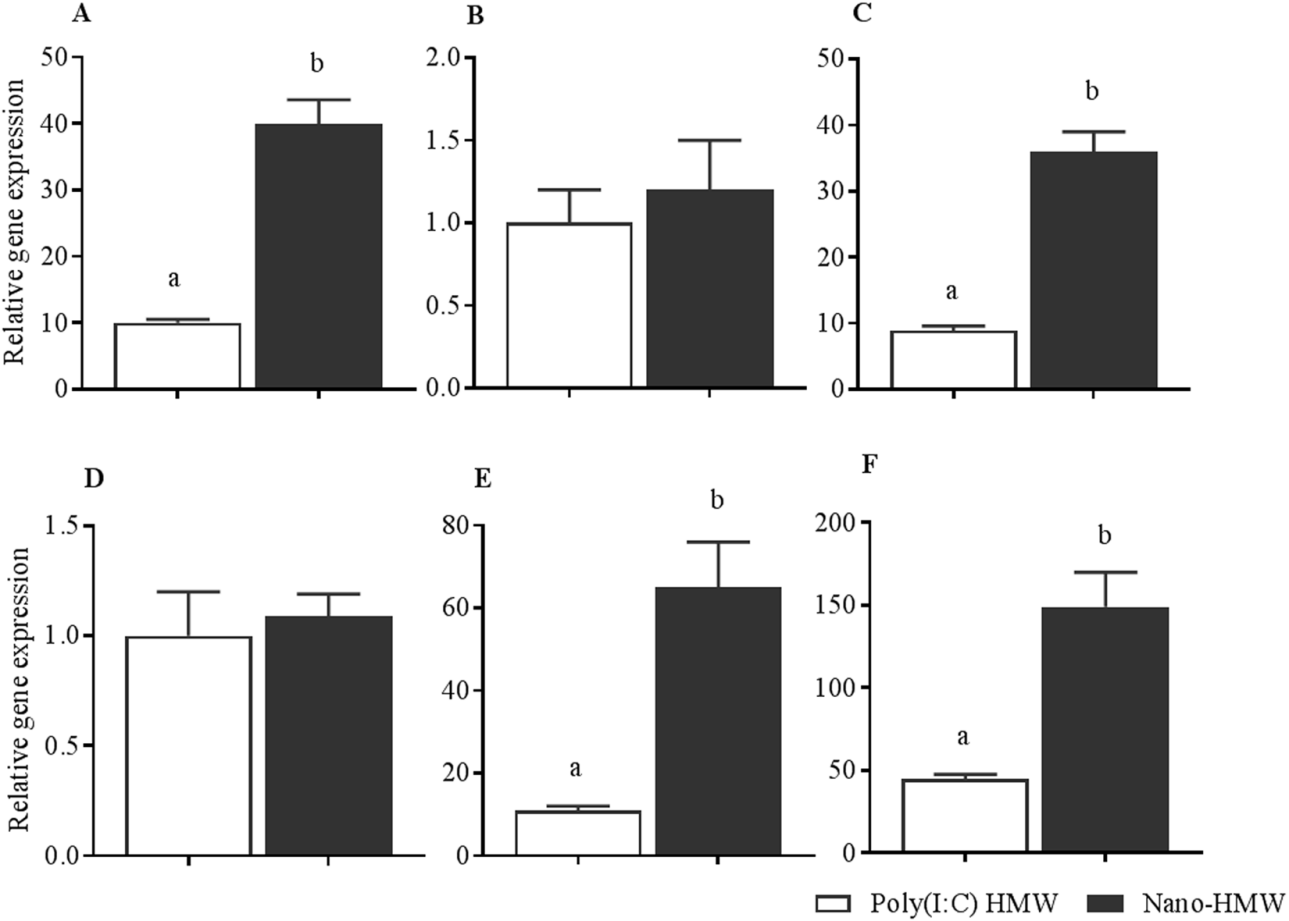
Innate antiviral gene expression in RTgutGC cell monolayer cultures induced with a low dose of HMW poly(I:C). Relative mRNA expression profiles in the respective order for IFN1, Mx1 and Vig3 were measured at 3 hr (**A**–**C**) and 6 hr posttreatment (**D**–**F**) in RTgutGC cell stimulated with 62 ng/mL HMW poly(I:C) or Nano-HMW. Media alone (mock) and cationic phytoglycogen NPs alone (Nano-mock) were included as controls and Nano-mock was not considered for comparisons as it did not induce any gene expression at any time point. The mean relative expression of IFN1 and ISGs in treated groups was compared to the mock treated group. Results are from three independent trials and data are shown as the mean ± SEM. *P* < 0.05 was considered significant using a paired Student’s T-test for data analysis. Different letters within a defined single time point show significant differences between groups.

**Figure 5:**
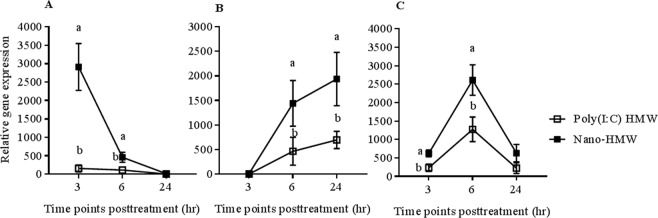
Innate antiviral gene expression in RTgutGC cell monolayer cultures induced with a high dose of HMW poly(I:C). Relative mRNA expression profiles of IFN1 (**A**), Mx1 (**B**) and Vig3 (**C**) in RTgutGC cell monolayers stimulated with 1000 ng/mL HMW poly(I:C) or with Nano-HMW. RTgutGC cells were treated for 3, 6 and 24 hr with media (mock), cationic phytoglycogen NPs (Nano-mock), HMW poly(I:C), and Nano-HMW. The mean relative expression of IFN1 and ISGs (Mx1 and Vig3) in HMW poly(I:C) and Nano-HMW treated groups were compared to the mock treated group. Results were from three independent trials and data shown are the mean ± SEM. *P* < 0.05 was considered significant using a two tailed Student’s T -test. Different letters within a defined single time point show significant differences between groups at that time point.

Next, dsRNA induced IFN pathways were investigated at the transcript level in RTgutGC cells grown in a monolayer culture, after stimulation with low (62.5 ng/mL) and high (1000 ng/mL) concentrations of HMW poly(I:C) for different amounts of time. IFN (IFN1) and ISG (Mx1 and Vig3) transcripts were measured by qRT-PCR (Figs 4 and 5). For the low dose treatment, at 3 hr, the expression of IFN1 was significantly upregulated by Nano-HMW (40 mean fold compared to medium alone) compared to by free HMW poly(I:C) (10 mean fold compared to medium alone) (Fig. 4A). Although there was no difference in expression of Mx1 between groups at 3 hr (Fig. 4B), the pattern of expression of Vig3 resembled that of IFN1, with Nano-HMW inducing a stronger response (36 mean fold compared to medium alone) compared to HMW poly(I:C) (8.9 mean fold compared to medium alone; Fig. 4C). At 6 hr posttreatment, the expression of IFN1 subsided in both groups (Fig. 4D); however, the expression of Mx1 (Fig. 4E) and Vig3 (Fig. 4F) remained significantly higher for Nano-HMW compared to HMW poly(I:C) alone. The Nano-HMW resulted in significantly higher Mx1 expression (65 mean fold increase compared to medium treated group), whereas the same dose of HMW poly(I:C) resulted in an 11-mean fold increase compared to media group. The mean fold increases for Vig3 expression with Nano-HMW and HMW poly(I:C) treatments were 149 and 45, respectively.

Next, differences in HMW poly(I:C) and Nano-HMW’s ability to induce IFN and ISGs were determined at a high concentration of dsRNA (1000 ng/mL; Fig. 5). Nano-HMW induced significantly higher IFN1 expression at 3 and 6 hr posttreatment compared to HMW poly (I:C) with a peak at 3 hr (2910 mean fold greater than the medium treated group). HMW poly(I:C) alone still induced substantially higher levels of IFN1 (156 mean fold increase compared to the media treated group), but significantly lower than Nano-HMW. Notably, the level of expression of IFN1 waned to lower levels at 6 and 24 hr posttreatment (Fig. 5A). With regard to ISGs, Nano-HMW enhanced Mx1 expression at later time points, with peak expression observed at 24 hr (Nano-HMW: 1936 mean fold greater than the medium treated group, poly(I:C) HMW: 698). At both 6 and 24 hr, Nano-HMW induced higher Mx1 transcript levels compared to HMW poly(I:C). However, no significant changes were observed at 3 hr posttreatment (Fig. 5B). Vig3′s peak expression was by 6 hr posttreatment (Nano-HMW: 2613 mean fold greater than the medium treated group; HMW poly(I:C): 1276). At 3 and 6 hr posttreatment, Vig3 expression was significantly higher in the Nano-HMW treatment group compared to free HMW poly(I:C). However, no significant differences in Vig3 expression were observed at 24 hr posttreatment between the two groups (Fig. 5C).

### Cationic phytoglycogen NPs enhance the antiviral effects of HMW poly(I:C)

**Figure 6:**
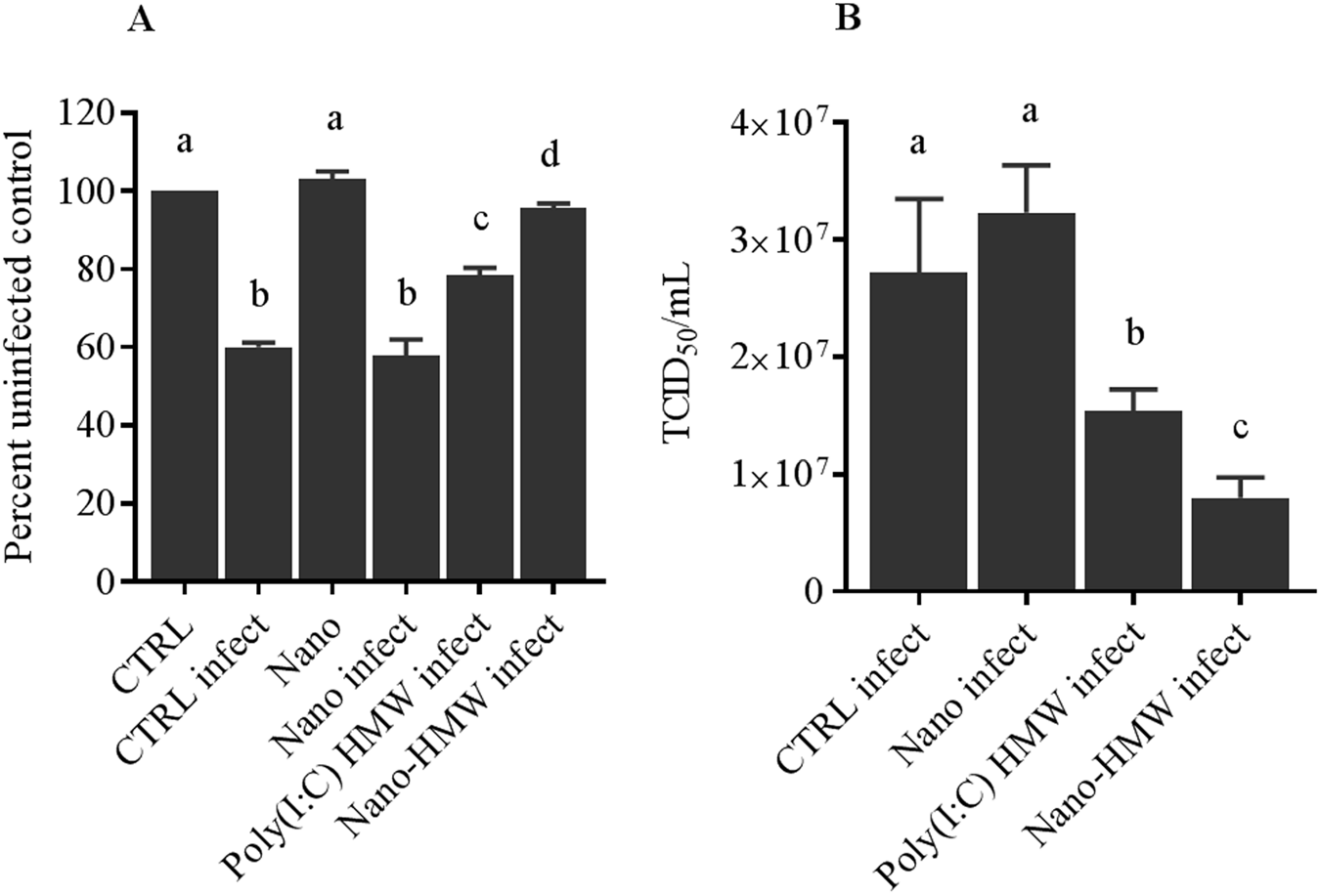
Determining the antiviral efficacy of free HMW poly(I:C) and Nano-HMW against VHSV-IVa. RTgutGC cells grown in a monolayer were infected with viral hemorrhagic septicemia virus strain IVa (VHSV-Iva; MOI = 1) 6 hr after treatment with HMW poly(I:C) or Nano-HMW at 1000 ng/mL. Controls included cells treated with media alone (CTRL), cells treated with media and infected by virus (CTRL infect), cells treated with NP alone (Nano-mock) and not infected (Nano) and infected (Nano infect). Cell viability was measured by Alamar Blue assay (**A**). Resulting virus titers from the cell supernatant were measured by TCID_50_ assay (**B**). Data are presented as mean ± SEM. Three independent trials were conducted. Data were analyzed by a one-way ANOVA with a Dunnett’s test. (*p* < 0.05). Different letters show significant differences between groups.

To evaluate whether the higher levels of IFN1 and ISG transcripts induced by Nano-HMW translated into enhanced protective antiviral activity, virus infection trials were performed. RTgutGC cells grown in a monolayer were treated with mock (media), cationic phytoglycogen NPs (Nano-mock), free HMW poly(I:C) (1000 ng/mL) and Nano-HMW (1000 ng/mL) for 6 hr prior to infection with VHSV-IVa (MOI = 1). Cell viability/intactness of the cell monolayer was assessed using the fluorescence indicator dye, Alamar Blue. Both free HMW poly(I:C) and Nano-HMW induced significant protection of the cell monolayer from infection; however, Nano-HMW provided significantly better protection compared to HMW poly(I:C) alone (Fig. 6A). Notably, the cationic glycogen NPs alone (Nano-mock or Nano) did not induce cellular toxicity nor protection to RTgutGC cells at 1000 ng/mL in serum free medium (Fig. 6A). Additionally, Nano-HMW effectively reduced virus replication in RTgutGC cells to a great extent compared to free HMW poly(I:C) and the control groups as measured by TCID_50_/mL (Fig. 6B).

### Cationic phytoglycogen NPs enhance the antiviral effects of HMW poly(I:C) in an *in vitro* intestinal cells and macrophage co-culture model

**Figure 7:**
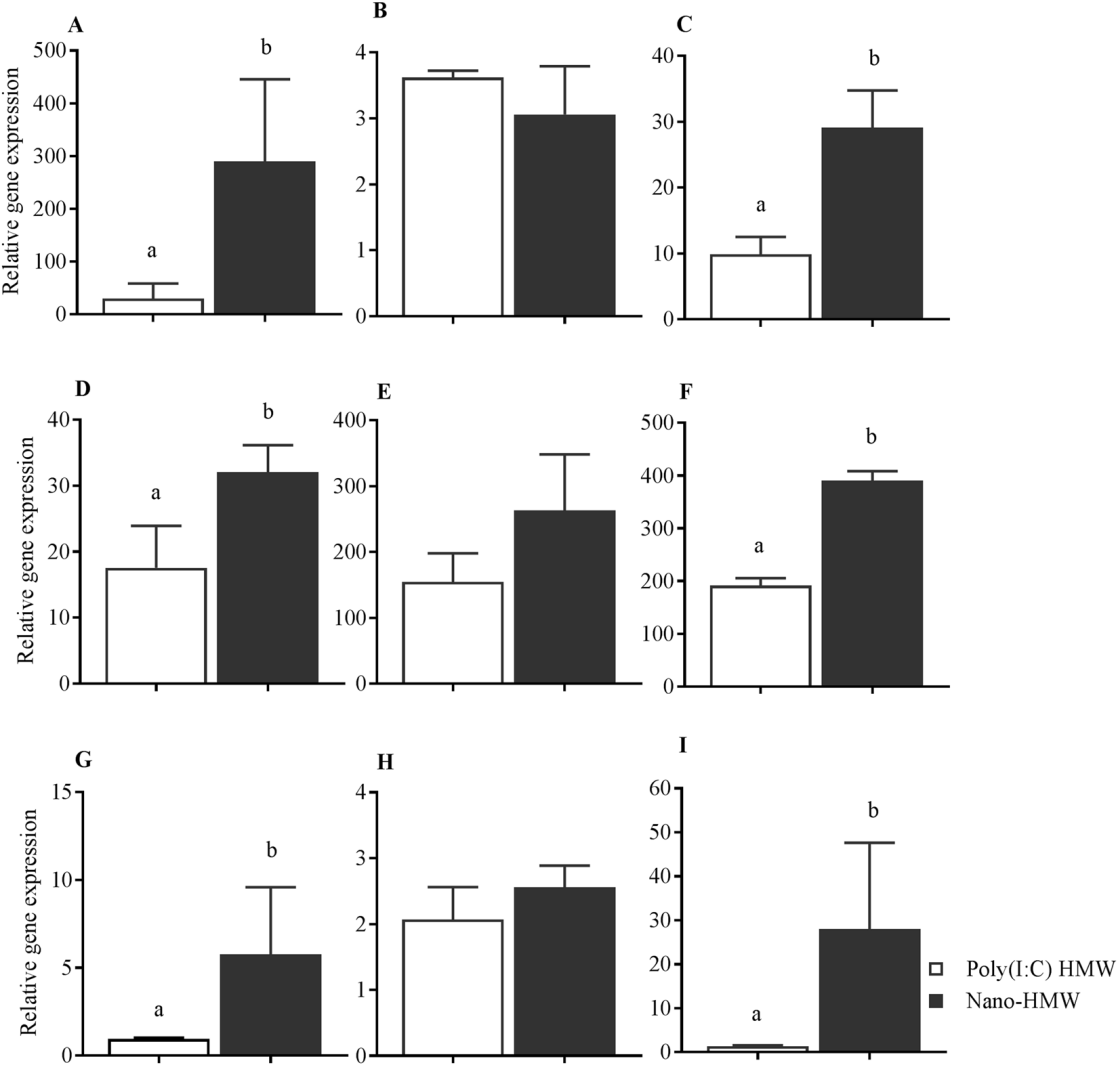
Innate antiviral gene expression *in vitro* by RTgutGC cells grown in transwell with or without an RTS11 co-culture. Relative mRNA expression profiles in the respective order for IFN1, Mx1 and Vig3 transcripts were measured at 3 hr (**A**–**C**) and 6 hr (**D**–**F**) posttreatment in RTgutGC cells cultured in a trans-well culture system. IFN1, Mx1 and Vig3 transcript levels were measured in RTS11 cells (**G**–**I**) co-cultured below RTgutGC cells grown in transwell inserts. Both culture systems were stimulated with 1000 ng/mL HMW poly(I:C) or Nano-HMW. For the co-culture experiment, RTS11 was plated in the lower well under the RTgutGC cell insert after 6 hr of stimulation of the RTgutGC cells from the apical surface. The mean relative expression of IFN1 and ISGs were compared to the cell culture medium treated group as the Nano-mock did not upregulate the expression of antiviral genes. Results were from three independent trials and data are presented as the mean ± SEM. *P* < 0.05 was considered significant using a two tailed Student’s T -test. Different letters within a defined single time point show significant differences between groups.

RTgutGC cells were cultured in transwell inserts to mimic an intestinal epithelium *in vitro*^26,27^. After the integrity and effectiveness of the tight junctions from RTgutGC cell transwell cultures were validated (Supplementary Fig. 1A,B), the expression of IFN1 and ISGs in response to stimulation with 1000 ng/mL HMW poly(I:C) and Nano-HMW was quantified. The relative expression of IFN1 at 3 hr poststimulation was significantly higher in the Nano-HMW (307 mean fold change compared to medium treated group compared to HMW poly(I:C) alone (17 mean fold change compared to medium group) (Fig. 7A). Similar to what was observed in the RTgutGC cell monolayers, there was no difference observed between treatments for Mx1 expression at 3 hr (Fig. 7B). The Nano-HMW resulted in significantly higher Vig3 expression (28 mean fold increase compared to medium treated group), whereas the same dose of HMW poly(I:C) resulted in a 10-mean fold increase compared to media group (Fig. 7C). At 6 hr, as the expression level of IFN1 waned (Fig. 7D) but was still higher in the Nano-HMW treatment compared to HMW poly (I:C). Mx1 transcript levels were enhanced in Nano-HMW but not statistically significantly different from HMW poly(I:C) alone (Fig. 7E). Vig3 attained significantly higher expression levels for Nano-HMW treatments compared to HMW poly(I:C) alone (Fig. 7F). In contrast to HMW poly(I:C), which resulted in a 190 mean fold increase in Vig3 expression compared to the medium group, Nano-HMW resulted in a 390 mean fold increase of Vig3 expression compared to the media group.

To determine whether an activated intestinal epithelial monolayer was able to transmit an antiviral signal to macrophages within the basal compartment, a co-culture of RTgutGC and RTSll was established. In this experiment RTgutGC cells were stimulated on their apical side with HMW poly(I:C) or Nano-HMW for 6 hr, after which RTS11 were seeded into the well compartment below the insert. RTS11 were then collected 3 hr post-seeding, and their antiviral gene transcripts were measured. RTS11 plated below RTgutGC cells treated with Nano-HMW demonstrated higher expression for IFN1 (Fig. 7G). The expression of IFN1 was a 6 mean fold increase in the Nano-HMW group and 0.95 mean fold increase for HMW poly (I:C). No difference was observed for Mx1 expression (Fig. 7H); however, RTS11 plated below RTgutGC cells treated with Nano-HMW demonstrated higher expression for Vig3 (Fig. 7I) compared to HMW poly(I:C) alone. Nano-HMW resulted in a 28-mean fold increase of Vig3 in RTS11 compared to medium alone, while  HMW poy(I:C) induced significantly lower Vig3 expression (1.3-fold mean increase compared to the medium group).

## Discussion

The specific activation of PRRs by ligands such as dsRNA has potential utility for a variety of therapeutic applications, including broad-spectrum antiviral immunotherapy and as vaccine adjuvants. However, a lack of efficient delivery systems for dsRNA in fish has hampered its practical application for controlling viral pathogens. To this end, a cationic phytoglycogen NP was used to deliver HMW poly(I:C), a model dsRNA molecule, and its ability to stimulate innate antiviral immune responses in rainbow trout cells was measured. Although delivering TLR ligands using NPs in fish is not a new concept, the NP used in the current study is unique. As opposed to most NPs, which have inherent immunotoxic or immunomodulatory activities within the host cells^33,34^, these phytoglycogen NPs were not able to induce innate immune responses on their own, nor did they induce cellular toxicity. These properties make this phytoglycogen NP a promising carrier for delivering immunomodulation agents in aquatic species.

NPs fabricated from lipid-derivatives, chitosan and poly(D,L-lactide-co-glycolic acid), with their inherent limitations listed above, have commonly been used to deliver TLR ligands such as dsRNA in fish^35–37^. Additionally, in fish the main route of TLR ligand or antigen administration is either by intraperitoneal or intramuscular injection, which results in enhanced innate immune responses^13,18^, but lacks practicality in the aquaculture industry. Ideally, triggering innate immune responses along mucosal surfaces would not only be more cost effective due to delivery ease, but would be more efficacious, preventing and blocking the pathogen at a primary site of infection^38^. Therefore, as a proof of concept, the induction of innate immune responses was tested *in vitro* using the rainbow trout gut epithelial cell line, RTgutGC cells. Innate immune responses were measured first in cells grown in a conventional monolayer and then cells were tested when cultured in a transwell system as well as in a co-culture system. The RTgutGC cell transwell system has been effectively used to mimic the gut epithelium in culture for feed development, immune modulation and toxicology purposes^26,27^. Thus, this model is ideal for initial *in vitro* studies to evaluate immune-stimulatory effects of TLR ligands in rainbow trout.

Before evaluating the immune modulation capacity of the Nano-HMW formulation, the physical characteristics of the NPs alone, bound to dsRNA, and associated with cells, needed to be elucidated. The results demonstrated that the phytoglycogen NPs were indeed cationic, approximately 70 nm in diameter and monodisperse. From the gel shift assay two conclusions can be made. Firstly, the NPs were able to adsorb dsRNA of different lengths, specifically lengths between 400–3000 bp. Secondly, at a ratio of 1:1, all dsRNA appeared to be adsorbed on the NP, with no free dsRNA detectable in the poly (I:C)-Nano lanes. Therefore, the effects observed from poly (I:C)-Nano in subsequent assays can be attributed to dsRNA associated with the NP and not free dsRNA. Additionally, the majority of RTgutGC cells were able to bind dsRNA and this binding was by an active mechanism, as more dsRNA was bound to cells at 20 °C compared to 4 °C. The binding pattern was punctate, which has been found previously in vertebrate cells^31^. It has been demonstrated previously that dsRNA binds rainbow trout cells via class A scavenger receptors (SR-As)^12,32^. The present work demonstrates that SR-As bind not only free HMW poly(I:C), but also Nano-HMW NPs as well.

The ability of Nano-HMW and HMW poly (I:C) to induce innate antiviral responses, evaluated in different cell cultures (RTG-P1, RTgutGC monolayer and transwell cultures, and co-cultures with RTS11), clearly indicated that in all systems tested, Nano-HMW induced a stronger innate antiviral response compared to HMW poly(I:C) alone. Importantly, while Nano-HMW induced higher levels of IFN1 and ISGs, negative regulation pathways remained intact; cells were able to reduce IFN1 and sometimes ISG expression after stimulation, over the first 24 h post treatment. Complementary to this data, RTG-P1 cells demonstrated less Mx1 promoter activity with each day, to 4d post treatment, further suggesting an intact negative regulation mechanism. This extended treatment assay also demonstrated that both treatments (HMW poly (I:C) and Nano-HMW) were able to induce significant Mx1 promoter activity up to 4d post treatment, suggesting the observed effects are long lasting. Long last effects could reduce administration frequency needed to protect animals *in vivo*.

The Nano-HMW-induced enhanced IFN1 and ISG expression resulted in greater protection of rainbow trout gut cells against viral hemorrhagic septicemia virus (VHSV-IVa) infection and reduced virus titres compared to HMW poly (I:C) alone. One explanation for why Nano-HMW was such a potent immune-stimulator may be that it induces more effective receptor clustering. Based on the dsRNA entry pathway proposed for mammalian cells^31^, fish SR-As may deliver dsRNA from the extracellular space into endosomes to be sensed by endosomal TLR3. TLR3 receptor clustering, due to high concentration or longer lengths of dsRNA, results in stronger downstream signaling pathways, leading to a stronger innate antiviral immune response^39^. Thus, Nano-HMW may be a stronger immune-stimulant because the dsRNA concentrated on the NP would be causing enhanced TLR3 receptor clustering compared to HMW poly (I:C) alone. This hypothesis has been exemplified in previous studies using nanoparticles for TLR ligand delivery. In one study, innate immune responses to CpG ODN directly corelated to the number of CpG per polygon particle^40^. A second study showed an increased density of CpG ODN on nanolipoprotein particles resulted in higher levels of activation on a per cell basis, as single cells encountered multiple copies of CpG ODN^41^. Additionally, self-assembling particulates displaying higher densities of TLR-7/8a agonist activated antigen presenting cells more effectively and increased innate immune responses^21^. Clearly, TLR ligands concentrated on a NP enhances host innate immune responses. Whether the degree of immunostimulation found in the current study correlates with the density of TLR ligands on the NPs is currently under investigation.

Additionally, it appears that immune responses generated within the intestinal epithelium is not restricted to this cell type but can be transmitted to underlying immune cells. The present *in vitro* transwell model data suggests that epithelial cells stimulated with Nano-HMW at the apical surface resulted in higher expression of soluble cellular factors on the basolateral surface that in turn induced antiviral gene expression in RTS11 macrophages cultured. These soluble factors may be IFNs, as IFNs in fish auto-amplify (i.e. IFN inducing expression of more IFN)^3^, a phenomenon that does not occur in mammals, but has been documented in avian macrophages^42^. It is possible that the innate immune responses measured in the intestinal cells cultured in the transwell and co-culture system may not directly translate into *in vivo* systems. Thus, future oral delivery studies in rainbow trout are being conducted to pursue whether the systemic effects suggested *in vitro* resemble those between the fish intestine and other internal organs.

In conclusion, surface adsorbed HMW poly(I:C) onto cationic phytoglycogen NPs triggered a stronger innate antiviral response at the cellular level compared to HMW poly(I:C) alone. As many next-generation vaccine technologies and broad-spectrum immunotherapies utilize the power of PAMPs, there is a great need to understand NP-mediated delivery systems for TLR ligands. The present study supports the use of cationic phytoglycogen NPs as carriers for TLR ligands in rainbow trout.

## Methods

### Cells

The RTgutGC cell line originated from the intestine of a female rainbow trout (*Oncorhynchus mykiss*) was kindly provided by Dr. Niels Bols (Biology Department, Waterloo University, Canada)^43^. RTgutGC cells were cultured in Leibovitz’s L-15 medium supplemented with 10% FBS and 1% P/S, expanded in 75 cm^2^ tissue culture flasks (BD Falcon) every 10 days. RTG-P1, a reporter cell line, was cultured in the same way as RTgutGC cells, however, Neomycin (G418, Sigma) was added in the media at a concentration of 200 μg/mL^44^. RTS11, a monocyte/macrophage cell line derived from rainbow trout, was cultured in Leibovitz’s L-15 medium supplemented with 20% FBS and 1% P/S and expanded in 25 cm^2^ tissue culture flasks (BD Falcon) every 3 weeks^45^. Epithelioma Papulosum Cyprinid (EPC) cells was cultured as described for RTgutGC cells. All cells were cultured at 20 °C, except EPC that was cultured at room temperature (~22 °C).

### Virus

Viral hemorrhagic septicemia virus strain IVa (VHSV-IVa) was kindly provided by Dr. Niels Bols. It was propagated on EPC cells with Leibovitz’s L-15 medium supplemented with 2% FBS and 1% P/S and incubated at 17 °C for 5–7 days. The virus preparation used in this study had a TCID_50_/mL of 2.15  × 10^8^/mL, as determined by the Reed and Meunch method^46^.

### Characterization of cationic phytoglycogen NPs and complex formation with HMW poly(I:C)

Phytoglycogen NPs obtained from sweet corn and commercialized under the tradename NanoDendrix™ (Glysantis^TM^, Guelph, ON, Canada) was used to prepare cationic phytoglycogen NPs. Briefly, a measured amount of phytoglycogen NPs was homogenized with a mixture of Glycidyltrimethylammonium chloride and NaOH in water. The reaction was heated at 45 °C for 4 hr and then neutralized and precipitated in 95% ethanol. The resulting cationic phytoglycogen NPs was pelleted, washed, dialyzed and freeze dried. The degree of substitution (DS) of hydrogens on the phytoglycogen NP’s hydroxyl **(**OH) groups for 3-(trimethylammonio)-2-hydroxyprop-1-yl groups that provide a net positive charge was determined by 1H-NMR spectroscopy in D_2_O at 348 K. The resulting preparation is identified to have a DS of 0.30. The physicochemical properties such as size and zeta potential (surface charge) of cationic phytoglycogen NPs were then characterized. The size and surface charge were determined by dynamic light scattering (Zetasizer Nano, Malvern Instruments, Worcestershire, UK). All measurements were conducted at 25 °C. HMW poly(I:C; catalog# tlrl-pic, HMW), a commercially available dsRNA, was obtained from InvivoGen and resuspended (1 mg/mL) in molecular biology grade water according to the manufacture’s recommendations. The cationic phytoglycogen NPs were dissolved in molecular biology grade water at 1 mg/mL. The complex between HMW poly(I:C) and cationic phytoglycogen NPs was formed at a ratio of 1:1 (w/w) assuming a theoretical loading of 100% (w/w), given that all HMW poly(I:C) is bound to the cationic phytoglycogen NPs. Briefly, 10 µL from 100 µg/mL cationic phytoglycogen NPs was mixed with 40 µL of 25 µg/mL HMW poly(I:C), pipetted to mix and incubated for 20 minutes at room temperature, allowing for electrostatic interactions to occur between negatively charged HMW poly(I:C) and cationic phytoglycogen NPs. The complex was run on a 1% agarose gel to determine the degree of HMW poly(I:C) incorporation onto the cationic phytoglycogen NPs using a gel shift assay. In this study, the HMW poly(I:C) cationic phytoglycogen NP complex is designated as Nano-HMW. Two poly(I:C) sources were tested for their ability to bind the cationic phytoglycogen NPs, denoted poly(I:C)^A^-Nano (Sigma-Aldrich) and poly(I:C)^B^-Nano (InvivoGen).

### Uptake of HMW poly(I:C) or Nano-HMW complex

For evaluating the uptake of the soluble HMW poly(I:C) and Nano-HMW, HMW poly(I:C) was labeled with Alexa Fluor® 546 using the Ulysis Nucleic Acid Labeling Kit (Invitrogen, Carlsbad, CA) according to the manufacturer’s instructions. After labeling the dsRNA was then complexed to the cationic phytoglycogen NPs. RTgutGC cells plated into 12 well-plates (2 × 10^5^ cells/mL) on glass coverslips and cultured overnight were treated with fluorescently labeled free HMW poly(I:C) or Nano-HMW (500 ng/mL) in serum free medium for 3 hr at 4 or 20 °C^12^. To examine whether the uptake of HMW poly(I:C) delivered either in soluble form or by nano-delivery differs in its entry mechanism, we compared the uptake of HMW poly(I:C) and Nano-HMW in the presence or absence of the class A scavenger receptor competitive ligand, poly I, and its non-competitive counterpart, poly C, (200 µg/mL). In this study, the cells were first treated with poly I and poly C (both from Sigma-Aldrich) for 3 hr and without washing the cells were treated with HMW poly(I:C) or Nano-HMW for the time points indicated above. The cells were washed 3 × with PBS and fixed with 10% neutral buffered formalin for 10 min at room temperature. Nuclei were stained with DAPI. Images were obtained by inverted fluorescence microscopy (Nikon Eclipse TiE with Qi1 camera) and analyzed using Nikon NIS elements. The fluorescence intensity was measured by automatic selection of the area surrounding DAPI stained nuclei, total number of cells within the image were counted, and the intensity/cell was calculated.

### Evaluating Mx1 transcriptional activity by luciferase reporter assay

RTG-P1 transgenic cells (derived from RTG2, rainbow trout gonadal cells) expressing firefly luciferase under the control of the trout Mx1 promoter^44^ was used to select appropriate doses of HMW poly(I:C) and Nano-HMW for subsequent *in vitro* experiments. Briefly, one day after plating (2.5 × 10^4^ cells/well) in a 96-well plate (BD Falcon) at 20 °C, RTG-P1 cells were treated for 24, 48, 72 and 96 hr with freshly diluted free HMW poly(I:C) or Nano-HMW (serially diluted (two-fold dilutions) from 1000 ng/mL to 0.9 ng/mL)). The cells were lysed and re-suspended in luciferase substrate. Luciferase activity was quantified as relative light units (RLU) using a Synergy HT plate reader (BioTek, Winooski, VT) using a luciferase cell culture lysis reagent (Promega Madison, WI) according to the manufacturer’s instructions. Data were normalized to total protein concentrations of the cell lysates. The inducibility was calculated per well by subtracting RLU obtained from samples treated with HMW poly(I:C) or Nano-HMW from samples left untreated (treated with media or cationic phytoglycogen NPs respectively (Nano- mock). The final results are presented as an average from three trials.

### RTgutGC cells grown in a monolayer on tissue culture plates

RTgutGC cells were plated into 12 well-plates (BD Falcon) with the density of 4 × 10^5^ cells/mL and cultured in Leibovitz’s L-15 medium supplemented with 10% FBS and 1% P/S overnight at 20 °C. The cells were then treated with two concentrations (1000 ng/mL or 62.5 ng/mL) of HMW poly(I:C) or Nano-HMW in serum and antibiotic free medium. At 3, 6 and 24 hr posttreatment, cells were collected in TRIzol reagent (Invitrogen) for RNA extraction.

### RTgutGC cells grown in a transwell culture system

RTgutGC cells were seeded at 62,000/cm^2^ on permeable PE Transwell cell culture inserts (24 mm diameter, 1 µm pore size; Corning; Elscolab) in Leibovitz’s L-15 medium supplemented with 10% FBS and 1% P/S and incubated at 20 °C^26^. The inserts were placed in 24-well cell culture plates (Corning; Elscolab), which delineate the upper (apical) and lower (basolateral) compartments that were filled with 100 µL and 600 µL of culture media, respectively. The media from both compartments were changed thrice in the first week, and weekly until 28 days post-seeding. The integrity and effectiveness of the epithelial tight junctions was verified by evaluating the paracellular diffusion of Lucifer Yellow (LY) CH lithium salt (457.2 Da; Molecular Probes). LY (500 µg/mL) diluted in Ca^++^/Mg^++^ PBS was added to the top chamber of the insert (0.1 mL) and 0.5 mL Ca^++^/Mg^++^ PBS was added to the bottom chamber. Cells were incubated at 20 °C for 1, 2, 4, 8 and 24 hr. The amount of LY in the top and bottom compartments was measured in a Synergy HT plate reader (BioTek, Winooski, VT, excitation, 485 nm; emission, 530 nm) along with a LY standard curve. Data were presented as percentage of the input LY on the top compartment^47^. Additionally, the transepithelial electrical resistance (TEER), a widely accepted technique to evaluate the integrity of tight junction dynamics^48^, was monitored over time by using an Epithelial Voltohmmeter (World precision instruments) according to the manufacturer’s instructions. One day before TEER measurements, complete fresh media was added on the apical side (to attain a total volume of 200 µL) and in the lower (basolateral) compartment for a final volume of 1.2 mL. After verifying tight junctions, cells were treated with 1000 ng/mL free HMW poly(I:C) or Nano-HMW. All treatments were conducted in serum and antibiotic free medium. At 3 and 6 hr of stimulation, cells were collected in TRIzol reagent (Invitrogen) for RNA extraction.

### RTgutGC cells and RTS11 co-culture

We explored the expression of antiviral response genes in RTS11 plated in the basolateral compartment following apical stimulation of RTgutGC cells, cultured on permeable PE Transwell cell cultures (cultured conditions as described above), with HMW poly(I:C) or Nano-HMW (1000 ng/mL). After the confirmation of tight junction formation (by LY migration and TEER measurements) the apical side of the RTgutGC cell transwell cultures were stimulated with HMW poly(I:C) or Nano-HMW for 6 hr, after which, RTS11 (6 × 10^5^/well) were added into the bottom wells in serum free medium. After 3 hr post-treatment, RTS11 cells were collected in TRIzol reagent (Invitrogen) for RNA extraction. Moreover, RTgutGC cells were collected in TRIzol at 3 and 6 hr posttreatment.

### Antiviral effects of HMW poly(I:C) in RTgutGC cells

RTgutGC cells (2 × 10^4^ cells/well) cultured into 96-well plates (BD Falcon) for 24 hr were treated with 1000 ng/mL of free HMW poly(I:C) or Nano-HMW in serum free medium. After 6 hr of pre-treatment, the cell monolayer was infected with VHSV-IVa at a multiplicity of infection (MOI) of 1 in Leibovitz’s L-15 medium supplemented with 2% FBS and 1% P/S. After 11-days of incubation, cell supernatants were collected, and virus titers were estimated by determining the 50% tissue culture infectious dose (TCID_50_) in EPC cells using Reed and Muench method^46^. The extent of cell monolayer destruction due to infection was determined by Alamar Blue (AB) assay as indicated by the manufacturer’s protocols (ThermoFisher Scientific).

### RNA extraction and real-time PCR

Total RNA was extracted from RTgutGC and RTS11 cells using TRIzol^®^ Reagent (Life Technologies, Carlsbad CA, USA) as per the manufacturer’s instructions. RNA was treated with Turbo DNA-free™ Kit (Invitrogen) to remove contaminating DNA. Complementary single-stranded DNA (cDNA) was synthesized from 500 ng of purified RNA using the iScript™ cDNA Synthesis Kit (Bio-Rad, Hercules, CA) following protocols provided by the manufacturer. The expression of antiviral response genes (IFN1, Mx1, Vig3) was detected by SYBR Green real-time PCR and quantified by the CFX Connect Real-Time PCR Detection System (Bio-Rad). The primers and PCR conditions are described previously^12,49^. The relative transcript levels of these target genes were normalized to trout β-actin and presented as fold changes over the untreated control group.

### Statistical analysis

All statistical analyses were conducted using GraphPad Prism (Version 7, GraphPad, La Jolla, CA). One-way analysis of variance with a Dunnett’s post-test was used to analyze viral titers and cellular integrity assessed by Alamar Blue. All data generated from gene expression studies, uptake and reporter assays were analyzed with a paired Student’s T-test and data were transformed when not normally distributed. Data are given as mean relative gene expression (±standard error of the mean) in the treated groups compared to the appropriate control group (either culture medium or cationic phytoglycogen NPs (Nano-mock) within a defined time point. A value of *p* < 0.05 was considered significant. The cationic phytoglycogen NPs (Nano-mock) did not up-regulate IFN1 or ISG gene expression *in vitro* and served as qualitative control and was not considered further in the analyses.

## Supplementary information

Dataset 1

## Data Availability

All materials, data and associated protocols will be promptly made available to readers by the authors upon request, with the exception of the nanoparticle, which will be provided promptly by Glysantis Inc. after the requesting individual signs a Non-Disclosure Agreement.
