# Supplementary material for: Enhancing innate antiviral immune responses in rainbow trout by double stranded RNA delivered with cationic phytoglycogen nanoparticles
Source: Sci Rep. 2019 Sep 20;9:13619. doi: 10.1038/s41598-019-49931-2 (PMC6754369; doi:10.1038/s41598-019-49931-2)
Supplement: Supplementary file 1 — Dataset 1 [file 41598_2019_49931_MOESM1_ESM.docx]

**Enhancing innate antiviral immune responses in rainbow trout by double stranded RNA delivered with cationic phytoglycogen nanoparticles**

Tamiru N. Alkie^1^, Jondavid de Jong^1,2^, Kristof Jenik^1^, Karl M. Klinger^2^ & Stephanie J. DeWitte-Orr^1*^

^1^Department of Health Sciences, Wilfrid Laurier University, Waterloo, ON, Canada

^2^Glysantis Inc., Guelph, ON, Canada

**^1^**^*^Corresponding

[sdewitteorr@wlu.ca](mailto:sdewitteorr@wlu.ca)


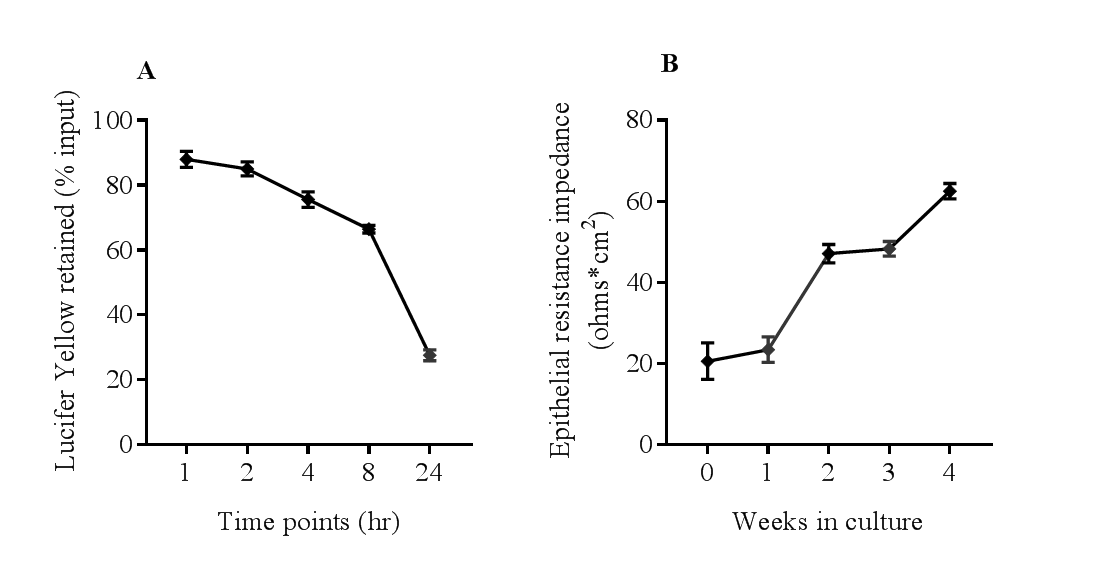
**Supplementary Figure 1.** Evaluating the integrity of RTgutGC cell monolayer and effectiveness of tight junctions following culturing of RTgutGC cells on transwell culture system. (A) Lucifer Yellow (LY) CH lithium salt (500 µg/mL) was added on the apical side and the amount of LY in the top and bottom compartments was measured. Data were presented as percentage of the input LY on the top compartment. (B) The transepithelial electrical resistance (TEER) was measured using Epithelial Voltohmmeter one day after complete media was added on the apical side (to attain a total volume of 200 µL) and in the lower (basolateral) compartment for a final volume of 1.2 mL.
